# Supplementary material for: Virtual triage and outcomes of diabetic foot complications during Covid-19 pandemic: A retro-prospective, observational cohort study
Source: PLoS One. 2021 May 6;16(5):e0251143. doi: 10.1371/journal.pone.0251143 (PMC8101747; doi:10.1371/journal.pone.0251143)
Supplement: Attachment — Submitted filename: Reply to Reviewers April 10.docx [file pone.0251143.s003.docx]

Sir,

I would like to thank you for your time during the ongoing COVID-19 pandemic giving an opportunity to revise the manuscript. I sincerely thank the editor and reviewers for their suggestions and constructive comments. We have revised the article as per your suggestions and provided the changes highlighted as blue in the revised manuscript.

Journal requirements:

1. Please ensure that your manuscript meets PLOS ONE's style requirements, including those for file naming. The PLOS ONE style templates can be found at

<https://journals.plos.org/plosone/s/file?id=wjVg/PLOSOne_formatting_sample_main_body.pdf> and

<https://journals.plos.org/plosone/s/file?id=ba62/PLOSOne_formatting_sample_title_authors_affiliations.pdf>

*Reply: The Requirement are checked, and revised manuscript is edited as per journal requirements.*

2. Please review your reference list to ensure that it is complete and correct. If you have cited papers that have been retracted, please include the rationale for doing so in the manuscript text, or remove these references and replace them with relevant current references. Any changes to the reference list should be mentioned in the rebuttal letter that accompanies your revised manuscript. If you need to cite a retracted article, indicate the article’s retracted status in the References list and also include a citation and full reference for the retraction notice.

Reply: Reference list is checked for accuracy in the *revised manuscript*.

3. We note that you obtained verbal consent from Group 1. In the Methods, please clarify whether consent was given to participate in this study or for another reason. Please also state in the Methods:
- Whether the Institutional Review Board (IRB) approved use of oral consent
- How oral consent was documented

For more information, please see our guidelines for human subjects research: <https://journals.plos.org/plosone/s/submission-guidelines#loc-human-subjects-research>

 Reply: Consent was given to provide tele consultation and participate in the study. The IRB approval is stated in the *revised manuscript*

4. In addition, in your ethics statement in the manuscript please ensure that you have discussed whether all data/samples from Group 2 were fully anonymized before you accessed them and/or whether the IRB or ethics committee waived the requirement for informed consent. If patients provided informed written consent to have data/samples from their medical records used in research, please include this information.

 Reply: The Data is anonymized in the electronic repository (group 2) and IRB approval is obtained. The same is mentioned in the *revised manuscript*

5. In your Methods section, please provide additional information about the participant recruitment method and the demographic details of your participants. Please ensure you have provided sufficient details to replicate the analyses such as descriptions of where participants were recruited and where the research took place.

 Reply: The same is added in the *revised manuscript as suggested.*

6. Please include the full name of the ethics committee that reviewed and approved your study in the manuscript Methods.

 Reply: The same is added in the *revised manuscript as suggested*

7. We note that you have indicated that data from this study are available upon request. PLOS only allows data to be available upon request if there are legal or ethical restrictions on sharing data publicly. For information on unacceptable data access restrictions, please see <http://journals.plos.org/plosone/s/data-availability#loc-unacceptable-data-access-restrictions>.

 Reply: The data is shared as supplementary file.

Editor Comments:

Please expand the table and figure legends including details like how data was represented (e.g. mean +/- standard deviation). Please provide values of statistical tests in addition to p value (e.g. odds ratio or other values). What was the power of study? Please also provide details about the human subject research approval of the study in the method part including the approval number and/or a statement indicating approval of this research. Also, the exclusion criteria of the patients should be presented as a separate section.

*Reply: The table and figure legends are expanded as detailed. Odds ratio were not calculated for independent group comparisons.*

*This was an observational retro-prospective study during the present ongoing COVID19 pandemic. Power of the study was not obtained.*

*Statement about research approval is provided in the revised manuscript (Method section)*

*Exclusion criteria are provided in separate section as suggested (Method section)*


Reviewers' comments:

Reviewer's Responses to Questions

**Comments to the Author**

1. Is the manuscript technically sound, and do the data support the conclusions?

The manuscript must describe a technically sound piece of scientific research with data that supports the conclusions. Experiments must have been conducted rigorously, with appropriate controls, replication, and sample sizes. The conclusions must be drawn appropriately based on the data presented.

Reviewer #1: Yes

Reviewer #2: Yes

*Reply: We thank you for your comments*

2. Has the statistical analysis been performed appropriately and rigorously?

Reviewer #1: Yes

Reviewer #2: Yes

*Reply: We thank you for your comments*

3. Have the authors made all data underlying the findings in their manuscript fully available?

The [PLOS Data policy](http://www.plosone.org/static/policies.action#sharing) requires authors to make all data underlying the findings described in their manuscript fully available without restriction, with rare exception (please refer to the Data Availability Statement in the manuscript PDF file). The data should be provided as part of the manuscript or its supporting information, or deposited to a public repository. For example, in addition to summary statistics, the data points behind means, medians and variance measures should be available. If there are restrictions on publicly sharing data—e.g. participant privacy or use of data from a third party—those must be specified.

Reviewer #1: Yes

Reviewer #2: Yes

*Reply: We thank you for your comments*

4. Is the manuscript presented in an intelligible fashion and written in standard English?

PLOS ONE does not copyedit accepted manuscripts, so the language in submitted articles must be clear, correct, and unambiguous. Any typographical or grammatical errors should be corrected at revision, so please note any specific errors here.

Reviewer #1: Yes

Reviewer #2: Yes

*Reply: We thank you for your comments*

5. Review Comments to the Author

Please use the space provided to explain your answers to the questions above. You may also include additional comments for the author, including concerns about dual publication, research ethics, or publication ethics. (Please upload your review as an attachment if it exceeds 20,000 characters)

Reviewer #1: The authors have submitted an interesting study wherein they have shown that teleconsultation with video call for visual inspection of the ulcers is very helpful in the care of Diabetic patients with foot ulcers or those who are at risk during the lockdown period necessitated by the Corona pandemic. They have shown that outcomes and wound healing rates were similar to a cohort of patients who had physical follow-ups in the same period a year earlier i,e. pre-lockdown. The authors have also discussed the limitations of this study because it would be applicable in a select subset of patients.

*Reply: We thank you for your comments*

Reviewer #2: The Manuscript “Virtual Triage and Outcomes of Diabetic Foot Complications during COVID-19 Pandemic: A Retro-Prospective, Observational cohort study” by Dr. Rastogi et al, Compares the care provided to patients with diabetic foot by virtual consultation during the COVID 19 pandemic and face-to-face care provided to the diabetic patients. It mainly focuses on the outcomes of diabetic foot and developing complications like foot ulcers in patients provided tele-consultations with those who attended foot clinic consultations in the pre-pandemic period. This research tried to elucidate the benefits of remote consultations over in-clinic consultations.

Allocation of patients in each group is appropriate for the study. Size of both groups is large enough for efficient statistical comparison of the treatment. Patient follow-up and wound assessment were performed by a single investigator, and trained personnel, would decrease the subjective error in the study. Because of the limitation due to COVID-19, investigations like difference in foot temperature and other DFU estimations are done by trained personnel at patients’ resident. Visual estimation of wound infection and wound healing, without appropriate investigations may limit the study but, author tried to address these limitations and overcome it by using the appropriate methods. Though the study duration is less, long enough to address the significance of the study. It would be more appropriate for author to include the parameters like HbA1c, FBG, PPBG and other complications like HTN and nephropathy at the end of the study. These parameters will reflect the patient’s compliance to diabetic treatment and give us the actual outcome of the foot ulcers.
This is manuscript is technically sound enough and the statistically significant data supports it.
I recommend that this paper be accepted.

*Reply: We thank you for your comments.*

*The detailed evaluation regarding nephropathy and hypertension was not possible during lockdown for patients in group 1 were provided virtual teleconsultations.*

*FBG and PPBG values were provided on home based self-monitoring of blood glucose in the revised manuscript.*

*HbA1c was not possible due to same reasons for lack of clinic visit.*

We hope that the revised manuscript may be considered for publication.

**Sincerely,**

**Ashu Rastogi**
